# Civil Malpractice

**Published:** 1873-06

**Authors:** 


### Books Reviewed.

*Civil Malpractice.* A Report presented to the Military Tract Medical Society at its Fifteenth Semi-annual Meeting, June 14th, 1873. By M. A. McClelland, M. D. Chicago: W. B. Keen, Cooke & Co., 1873. 800 pp. Price $2 00.

Dr. McClelland's Report is full of interest, and considers a snbject which is of much importance, especially to those who are engaged in an active surgical practice. The question as to what may be held to be malpractice by the courts is of no inconsiderable interest to all who are subject to these troublesome and vexatious suits.

The term civil malpractice is construed to mean "an improper discharge of professional duty, either through want of skill or through negligence." "Thus, the improper performance of work requiring skill and knowledge, in any profession or business, would be designated 'malpractice,' and laws bearing upon it would be the same." As one of the reasons why more is expected from physicians and surgeons than from others the author gives the following: "In respect to surgical cases that are investigated in our courts, how frequently we hear surgeons—experts—boasting of the cures they have effected in similar cases; no shortening, no deformity, in their experience; leading the courts, leading juries, leading every one within the sound of their voice to suppose that perfect cures were and should be the rule, and imperfect cures the exception."

That this is often the case there can be no doubt in the minds of those who have witnessed the proceedings in cases of malpractice. Physicians, either through carelessness, or a desire to magnify themselves and their cases, before court and jury, seem to forget the fact that all are at times liable to failure, and that the peculiar characteristics of the patient, and the circumstances of time and place are to be all taken into consideration in judging of any particular case. No one case can be set up as a standard by which to judge others. The law however seems to be with the physician, and no surgeon, if he has acted in accordance with his best knowledge and skill, need fear to have his acts investigated if, and here comes the turning point of most suits for malpractice, he can get a jury who will not allow their feelings to influence their decision. The defendant in a case of malpractice may be, for instance, possessed of sufficient means to tempt the cupidity of some dissatisfied patient, together with some shyster of a lawyer, plenty of whom, we are sorry to say, can be found to prosecute a case on promise of half the spoils. These make up a case, which they carry before the jury with a piteous plea concerning the patient's poverty, the cruelty and negligence with which he has been treated, and the

*immense* wealth of the defendant, which renders him abundantly able to pay the damages claimed. The case is tried, witnesses on both sides show that the case has been treated with proper care and diligence. The Judge's charge is fair and impartial, and yet in not a few cases the physician is surprised by a verdict against him. Some member of the jury even having the boldness afterward to admit to the doctor that the case was treated all right, yet that they thought that he was able to pay the poor fellow something, and as he seemed sick or disabled they rendered their verdict in his favor. We are not overcoloring the picture, and could, did we think proper, give illustrative cases. Juries can and have been found who, in direct violation of their oath, will bring in a verdict to suit themselves; witness the confession of the jury in the Stokes case.

Dr. McClelland quotes from many different sources decisions of the courts in cases of malpractice, which show that the courts are not inclined to deal at all harshly with medical men, but to allow them all the law will permit in their several cases. Many of the decisions quoted are very interesting and we should be happy to place them before our readers, but can only recommend them to read Dr. McClelland's work. It is highly instructive and interesting.

